# Agreement between Myocardial Infarction Patients and Their Spouses on Reporting of Data on 82 Cardiovascular Risk Exposures

**DOI:** 10.1371/journal.pone.0132601

**Published:** 2015-07-10

**Authors:** Hedley Knewjen Quintana, Max Vikström, Tomas Andersson, Johan Hallqvist, Karin Leander

**Affiliations:** 1 Institute of Environmental Medicine, Karolinska Institutet, Stockholm, Sweden; 2 Center for Occupational and Environmental Medicine, Stockholm County Council, Stockholm, Sweden; 3 Department of Public Health and Caring Sciences. Uppsala University, Uppsala, Sweden; Universite de Montreal, CANADA

## Abstract

**Background:**

The validity of exposure data collected from proxy respondents of myocardial infarction patients has scarcely been studied. We assessed the level of disagreement between myocardial infarction patients and their spouses with respect to the reporting of the patient´s cardiovascular risk exposures.

**Methods:**

Within the frame of the Stockholm Heart Epidemiology Program (SHEEP), a case-control study of risk factors of myocardial infarction performed in Stockholm county 1992–1994, a subset of 327 first time myocardial infarction cases aged 45–70 who survived >28 days after the event and who co-habited with a spouse or common-law spouse (proxy) were identified between 1993-04-05 and 1993-12-31. Among these, 243 cases participated along with their respective proxy in the present study. Control individuals, matched to cases by age, sex and residential area were also included (n = 243). Data were collected using questionnaires. Using conditional logistic regression we calculated for each of 82 exposures the odds ratio based on information collected from 1) myocardial infarction cases and controls [odds ratio A] and 2) proxies and the same set of controls [odds ratio B]. Disagreement was measured by calculating the ratio between odds ratio B and odds ratio A with 95% confidence intervals (CI) calculated using resampling bootstrap.

**Results:**

For the vast majority of the exposures considered including diet, smoking, education, work-related stress, and family history of CVD, there was no statistically significant disagreement between myocardial infarction patients and proxies (n = 243 pairs). However, leisure time physical inactivity (proxy bias = 1.59, 95% CI 1.05-3.57) was overestimated by spouses compared to myocardial infarction patients. A few other exposures including some sleep-related problems and work-related issues also showed disagreement.

**Conclusions:**

Myocardial infarction patients and their spouses similarly reported data on a wide range of exposures including the majority of the traditional cardiovascular risk factors, leisure time physical inactivity being an exception.

## Introduction

Ischemic heart disease is the leading cause of premature death worldwide [1]. Although the knowledge about risk factors for myocardial infarction (MI) has increased over the years, there is still a need to learn more about MI aetiology to prevent morbidity and mortality. Studies with case-control design are important in examining how different exposures influence risk of MI. Any case-control study that evaluates the aetiology of a disease with a rapid evolution as outcome, such as MI, may require the collection of data from next-of-kin or other proxy respondents in order to determine exposure in the most severe or fatal cases. In case-control studies of MI, fatal cases are usually excluded and thus results can only be generalized to non-fatal events. However, about 28% of first MI events are fatal (death within 28 days) [2] and characteristics and risk factor distribution in this group may differ from that in non-fatal cases.

Earlier studies that have evaluated the validity of data collected from proxy respondents often focused on data collected from spouses to index individuals because researchers have assumed co-habitants are well informed about each other’s habits. In general, spouses also have higher response rates as compared to other types of proxy respondents [3–6]. The validity of exposure data collected from proxy respondents of MI patients has previously only been studied regarding alcohol intake: a study from Auckland, New Zealand reports a fairly good agreement between MI patients and their next-of-kin (especially spouses) with respect to reporting of drinking habits [7]. Results from earlier validation studies where cases had a cardiovascular disease diagnosis other than MI suggest a high level of agreement between spouses on the reporting of body size [3] and previous medical conditions [3, 4]. A moderate level of agreement, however, was observed for the reporting of leisure time physical activity [4].

The aim of the present study was to increase knowledge about the general validity of data collected from proxy respondents to MI cases. Such knowledge is crucial for the planning and choice of design of future epidemiological studies of MI that aim to consider also fatal events (and not only non-fatal events). We addressed the research question of whether there is disagreement between MI patients and their spouses with respect to self-reported data on cardiovascular risk exposures. The data we used came from the Stockholm Heart Epidemiology Program (SHEEP), a population-based case-control study designed to study risk factors for MI in men and in women. From a vast amount of exposure data collected in the SHEEP, eighty-two exposure variables representing 6 different areas of exposures were selected for inclusion in the present study based on their level of cardiovascular epidemiology relevance. We had no specific hypothesis regarding disagreement for any of the variables included. This study is unique in assessing such a large range of exposures. The design of our study followed the recommendations in Guidelines for Reporting Reliability and Agreement Studies (GRRAS)[8]

## Material and Methods

### Study population

This validation study is based on a subset of individuals included in the SHEEP, a case-control study of risk factors for MI performed in Stockholm County among men and women 45–70 years old who were Swedish citizens and free from previous MI. Details about the SHEEP design have been described in an earlier study [9]. Briefly, first time MI events were identified in the greater Stockholm area between 1992 and 1994. Over the study period, control individuals (at least one control per case) were continuously (within 2 days from case incidence) and randomly selected from the study population using the Stockholm county population register after matching for sex, age (five-year intervals), and residential area. All cases and controls completed a questionnaire, which included questions about life style, body habitus, environmental exposures, and psychosocial environment. The questions to cases and controls were almost identically formulated. The median time to response among cases was 26 days (interquartile range, IQR: 17–38 days), and among controls it was 39 days (IQR: 26–93 days).

Eligible cases for this study were non-fatal MI cases (survival after MI for at least 28 days) participating in the SHEEP and who received the questionnaire between April 5^th^ 1993 and December 31^st^ 1993. In addition, these cases were co-habiting with a spouse or a common-law spouse at the time of the MI event. After each eligible case was identified, the SHEEP secretariat mailed to each eligible case a letter of invitation. The letter included information about the study and asked for permission to contact his/her spouse/common law spouse (proxy). If the case agreed to participate, the proxy was sent a similar letter of invitation. Eligible proxies were also contacted by telephone in order to provide additional information about the study and to seek informed consent to participate. The proxies who agreed to participate were asked to complete a questionnaire similar to the one the MI patients completed. The proxies were asked to complete the questionnaire without help from their spouses, the MI patients. Participants who left questions unanswered were contacted by telephone and were asked the questions again. When the data collection had ended, the proxies were sent a letter where they were asked about whether some parts of the questionnaire were difficult to fill out and whether they received any help from their spouses, the MI patients, in responding to any of the questions. None of the proxies reported they received help from their spouses. However, 5 reported they had some difficulty giving information about work related exposures.

Among the 480 non-fatal cases who participated in the SHEEP during the study period, 327 were eligible for inclusion. Out of these, 31 either changed their marital status or died before the proxy was recruited in the present sub-study. Among the 296 remaining eligible cases, 53 proxies did not participate. Thus, the analyses are based on 243 case-proxy pairs.

The median time between when the cases completed their questionnaires and their respective proxies completed their questionnaires was 13 months (range: 4–24 months).

The controls were approached in a similar way as were cases and proxies, with invitation letter, questionnaire and telephone reminder if needed.

### Ethics statement

The study was approved by the Ethical Committee at Karolinska Institutet (91:259). All study participants gave their informed oral consent to be enrolled in the study; at the time the study was initiated (1992) forms for written consent were generally not used. An invitation letter was sent by mail to eligible subjects informing about the study. The information included description of rationale for the study, study aims, study design and that participation involved filling out a questionnaire. The letter also stated that participation was voluntary and that confidentiality was guaranteed. The receiver of the invitation was asked to return the completed questionnaire by mail. Employees at the SHEEP central received the questionnaires and contacted each respondent by telephone, asking about clarification of unclear answers and documenting informed consent to participate. A second letter of invitation, a reminder, was sent to those who did not reply to the first letter, and the same procedure for documentation of oral consent was used.

### Variable definitions

Traditional cardiovascular exposures. Hypertension was considered present if the participants answered “yes” to the question “do you have hypertension?”/”does your close relative have hypertension?”, or reported use of any medication against hypertension. Identification of diabetes and hyperlipidaemia, respectively, was correspondingly performed.

Based on self-reported data on weight and height we calculated the body mass index (BMI). Overweight was defined as a BMI of 25 kg/m^2^ or more whereas obesity was defined as a BMI of 30 kg/m^2^ or more.

Current smoking of cigarettes, cigars, cigarillos or pipes or cessation of smoking within the last two years was classified as current smoking. Stopping smoking more than two years ago was classified as former smoking. Never having smoked regularly for at least 1 year was classified as never-smoking. Daily use of moist snuff/Swedish tobacco in the preceding year was classified as use of smokeless tobacco (as opposed to non-use).

Physical inactivity was defined based on reports about level of leisure time physical activity in the previous 5–10 years. Reports of “very little exercise” or “isolated walks only” were considered exposed. The reference category includes “regular exercise (at least once weekly)” and “exercise once in a while”.

The sitting time was asked in relation to daily working hours in the previous 5–10 years. Reports of “almost all the working hours” were considered exposed. Reports of “half of the working hours” or “less than half of the working hours” were considered unexposed.

Previous non-MI CVD was defined as the presence of any of the following conditions: heart failure, stroke, angina and intermittent claudication.

Based on answers to questions with predefined answer alternatives about diagnoses and causes of death (if relevant) in parents and siblings before they turned 65 years old, participants were classified into categories of maternal, paternal and sibling history of CHD and CVD respectively. CHD comprises sudden death, MI and angina whereas CVD comprises CHD and stroke. “Don’t know” was also included as a predefined answer alternative.

Our analyses used different approaches: A) Considering individuals who provided full information about history of disease and cause of death (if relevant) in the parent and potential siblings, while excluding individuals reporting “don’t know” to either of these questions. B) Considering individuals who provided any information about history of disease or cause of death (if relevant) in the parent or potential siblings. In approach B, “don’t know” answers were set to “no”.

#### Dietary habits, intake of alcohol, coffee and vitamin supplements

The participants were asked to report the average daily or weekly intake of the following dietary items in the preceding year: 1) Fruit and berries, 2) Roots and vegetables (except potatoes), 3) Meat and sausage dishes, 4) Fish dishes and 5) Use of shortening, cooking oil and sauce, respectively. The number of servings per occasion was asked for as well as how often the dish was on the menu. In order to have the total number of servings per week we multiplied the number of times per week reported by the number of daily servings reported for each dietary item. We considered as exposed those who reported frequency of intake above the median level of intake in the group of control individuals. The following cut-off levels were used: 1) Fish, one serving per week; 2) Use of shortening, cooking oil and sauce, 4 servings per week; 3) Meat and sausages, 3 servings per week; 4) Roots and vegetables, 6 servings per week and 5) Fruits and berries one serving daily. Study participants who reported coffee drinking were asked how many cups consumed either weekly or daily in the preceding year. We calculated the number of cups daily and chose a cut-off of 3 cups daily (based on the median consumption of coffee among controls) for exposure to high coffee consumption. The intake of less than 3 cups daily was used as reference category.

The regular intake of vitamin supplements: vitamin supplements, minerals or other dietary supplements were assessed simply from answers to a yes/no question regarding intake in the preceding year.

For the present study, we consider information about frequency of intake of light beer, strong beer, wine and hard liquor in the preceding year as well as in the preceding 5–10 years. In addition, the serving sizes of each alcohol beverage (number of cans, glasses or bottles consumed at each drinking occasion) were considered.

For the reporting of frequency of light and strong beer intake in the preceding 5–10 years, four pre-defined categories were given: 1) Never, 2) One or two cans/bottles per week, 3) Three to nine cans/bottles per week and 4) Ten cans/bottles per week or more. For the reporting of wine and hard liquor consumption in the preceding 5–10 years, the predefined categories were: 1) Never, 2) Once monthly, 3) Every week and 4) Every day. For classifying individuals as exposed to high intake of each specific beverage, we used as cut-offs the median frequency of intake in the distribution in controls. The cut-off for both light and strong beer was 3 cans/bottles per week or more. For both wine and hard liquor the cut-off was “Drinking every week or more often”.

Regarding serving sizes of wine and hard liquor in the preceding 5–10 years, the following predefined answer alternatives were given: 1) More than one glass, 2) A couple of glasses, 3) Half a bottle and 4) One bottle or more. The median values of the controls´ distributions were used as cut-off. Participants classified as exposed to large serving size of wine and hard liquor in the preceding 5–10 years, respectively, were those who reported a serving size of half a bottle or more.

Based on reports about frequency of intake of each of the alcohol beverages in the preceding year, as well as information about serving sizes, we calculated the average daily alcohol consumption in grams. Participants with values above the median value of the distribution in controls (10.02g) were considered exposed to high alcohol consumption. Participants with values of 10.02g or lower were considered unexposed. The latter group includes participants reporting no alcohol intake.

#### Work-related factors

Job strain was determined using the Swedish version of the demand-decision latitude questionnaire implemented in SHEEP [10]. The subject reported the average situation in the preceding 5 years. The demand sum score, the decision latitude sum score and their ratio were calculated for each subject. The 75th percentile of this ratio amongst all the controls in the SHEEP study (0.765) was used as the cut-off value to define exposure to job strain according to the “Quotient job strain model”; subjects with a score above the cut-off value were classified as exposed to job strain and all other respondents were considered unexposed.

Binary variables were created for having subordinates, working shifts, receiving a monthly salary and having previously been unemployed.

Exposures to different pollutants at workplace: motor vehicle gases, particulate matter, combustion by-products, lead and dynamite were assessed using 3 possible answers: “yes”, “no” and “don’t know”. Those who reported presence of any of the pollutants were considered exposed to pollution at workplace. Those who answered “no” to all pollutant items were considered unexposed, whereas individuals answering “don’t know” were excluded from the analysis.

Psychosocial factors. The economic situation of the family during childhood was assessed from questions about economic problems before the age of 16. Three predefined answer alternatives were given: 1) No economic problems important enough to mention, 2) Small and/or reasonably short-term economic problems, and 3) Severe and/or long-term economic problems. We created a variable called “Economic problems before age 16” where answer alternatives 2 and 3 were considered exposed. A variable called “Severe economic problems before age 16” was also created, where answer alternative 3 was considered exposed whereas answer alternative 1 and 2 were considered unexposed.

Based on the highest educational level reported by the participants, three categories were formed: 1) Compulsory education (9 year of education), 2) Complete high school (12 years of education) and 3) University (more than 14 years of education). For the analysis of compulsory education we considered as exposed those belonging to that category; other responses were considered unexposed. In the same fashion, for the university education we considered as exposed those belonging to that category; other responses were considered unexposed.

For the following stressful events, if they occurred within a year before the survey, binary variables were created: 1) Conflict with spouse, 2) Death of relative or friend, 3) Disease/accident in spouse, 4) Death of a close relative/friend, 5) Impaired personal finances, 6) Conflict at workplace, 7) Moving, 8) Change of job, 9) Decreased responsibility at work and 10) Increased responsibility at work.

Four questions related to coping strategies were posed to participants: 1) “How often did you feel you could not control important matters in your life?”, 2) “How often did you feel confident in your ability to handle your personal problems?”, 3) “How often did you feel things turn out the way you wanted?” and 4) “How often did you feel you could not manage the difficulties?” For each item, there were five possible responses: 1) “never”, 2) “almost never”, 3) “sometimes”, 4) “often” and 5) “very often”. We dichotomized those ordinal variables according to the median among controls: We considered as exposed those who answered “often” or “very often” in the questions 1 and 4. For questions 2 and 3 we considered exposed those who answered “sometimes”, “often” or “very often”. In all the four items, all who answered any other response were considered unexposed.

#### Sleep-related problems

The participants were asked to report how often (considering the previous year) they had the following sleep-related problems: 1) Difficulties to fall asleep, 2) Difficulties to wake up, 3) Difficulty to go back to sleep, 4) Heavy snoring, 5) Nightmares, 6) Not feeling thoroughly rested after waking up, 7) Waking up too early, 8) Restless while sleeping, 9) Feeling tired/sleepy, 10) Eyes tired/irritated, 11) Experiencing non-voluntary falling asleep at workplace, 12) Experiencing non-voluntary periods of sleep during leisure time. 13) Feeling fatigued and easily distracted.

For each sleeping problem item, there were five predefined answer alternatives: “never”, “a few occasions yearly”, “a few occasions monthly”, “a few occasions weekly” or “on daily basis”. Based on the distributions of controls, we classified those reporting at the median or above as exposed; for all sleeping-related problems except from number 12 (see above), the answer alternative “a few occasions monthly” was the median. For number 12, it was “a few occasions yearly”.

### Statistical analysis

We calculated the prevalence of each exposure according to data collected from cases, proxies, and controls. To analyse possible bias introduced by using data collected from proxies instead of cases, we calculated two odds ratios (ORs) using conditional logistic regression: 1) OR-A from a model based on exposure data collected from cases and their matched controls, and 2) OR-B from a model based on exposure data collected from the proxies and the same set of controls. For each exposure, we calculated the quotient between OR-B and OR-A. We refer to this quotient as “proxy bias”. A proxy bias of 1.0 indicates that on average proxies do not systematically overestimate or underestimate the exposure in question as compared to the estimates provided by their paired MI case. Values below 1.0 indicate that proxies underestimate the exposure and that OR-B is underestimated. Correspondingly, values above 1.0 indicate that proxies overestimate the exposure and that OR-B is overestimated. We calculated 95% confidence interval (CI) boundaries of the proxy bias using resampling bootstrap based on 5 000 iterations, considering the difference between the variable coefficients of the model producing OR-A and OR-B. The bootstrap is suitable because it is based on very limited assumptions about the probability distribution that gave rise to the data [11]. Logistic regressions and the bootstrap analyses were performed using SAS version 9.2.

As far as we know, this method to assess disagreement between cases and proxies has not earlier been used. Compared to traditional methods that assess agreement, such as the kappa statistic [12], our method is quite different, as it gives the direction of disagreements (over- or underestimation by proxies).

Because several different approaches to analyse our data are possible, we provide the raw data (S1 Dataset) forming basis for the calculations of additional estimates of agreement between cases and proxies, such as sensitivity, specificity, positive predictive value, negative predicted value, kappa value, perfect agreement and marginal homogeneity (McNemar´s test).

## Results

### General description

The proxies reported that they had known their respective index case for 33.4 years on average (standard deviation 11.6 years). Only three proxies reported they had known their respective index case for less than five years. The mean time of the relationship was four years longer in female cases than in male cases.

The mean age of index cases was 59.5 years (standard deviation 6.9 years); the mean age of proxies was 57.0 years (standard deviation 9.1 years). Compared to the other non-fatal cases included in the SHEEP during 1992, the cases included in the present sub-study have similar distributions of sex, age, and residential area. Selected population characteristics are presented in Table 1.

**Table 1:** Selected population characteristics of the subset of SHEEP cases and controls included in the present validation study.

Variable	Cases N = 243	Controls N = 243	p value
Male sex	198 (81.5%)	198 (81.5%)	1.0
Age in years (continuous)^a^	59.5±6.9	59.5±6.9	1.0
Age (discrete)			
45–50	34 (14.0%)	34 (14.0%)	
51–55	35 (14.4%)	35 (14.4%)	
56–60	61 (25.1%)	61 (25.1%)	
61–65	52 (21.4%)	52 (21.4%)	
66 or more	61 (25.1%)	61 (25.1%)	1.0
Systolic blood pressure (mmHg)^a^	131±20	141±22	<0.01
Diastolic blood pressure (mmHg)^a^	80±10	83±10	<0.01
BMI^a^	26.6±3.5	25.4±3.6	<0.01
Serum HDL cholesterol (mmol/l)^a^	1.09±0.28	1.28±0.33	<0.01
Serum LDL cholesterol (mmol/l)^a^	4.17±0.94	3.98±1.00	<0.01
Serum total cholesterol (mmol/l)^b^	6.22 (4.78;7.52)	3.93 (2.85;5.18)	<0.01
Serum triglycerides^b^	2.18 (1.03;3.52)	1.20 (0.70;2.68)	<0.01

Counts and proportions are shown for categorical variables.

^a^Mean value ± standard deviation.

^b^The 50th percentile value with the 10th and 90th percentile values within parenthesis.

p values were calculated using chi square test, t test or Kruskal-Wallis test as appropriate.

Distributions of the cardiovascular risk exposures under study in cases, proxies and controls are presented in Table 2 and S1 Table. Overall, the traditional cardiovascular exposures were more frequent in cases than in controls (Table 2). In general, the prevalence of the variables in this study is above 10% (Table 2 and S1 Table), as reported by either cases or proxies, with some exceptions. A prevalence below 10% was noted for stroke, intermittent claudication, heart failure, some specific job-related exposures, and some important life events (S1 Table). For some of these variables, the bootstrap analysis was inefficient and no stable results could be obtained. For these variables, marked in S1 Table, the proxy bias estimate is not shown.

**Table 2:** Information forming basis for assessment of disagreement between cases and proxies on the reporting of traditional cardiovascular exposures.

Variable	Case Response	Proxy Response		Number of pairs	Prevalence (%), case data	Prevalence (%), proxy data	Prevalence (%), control data	Kappa	OR-B	OR-A
		Yes	No						95% CI	95% CI
Hypertension	**Yes**	75	12	238	37	35	23	0.90	1.80	1.72
	**No**	9	142						1.22–2.66	1.16–2.55
Diabetes	**Yes**	27	3	241	12	12	5	0.98	2.76	2.61
	**No**	1	210						1.40–5.59	1.30–5.26
Hyperlipidaemia	**Yes**	70	3	144	51	58	33	0.83	2.64	3.64
	**No**	14	57						1.53–4.56	2.06–6.47
Leisure time physical inactivity	**Yes**	77	20	237	41	53	36	0.63	1.26	2.00
	**No**	49	91						0.87–1.83	1.38–2.91
Personal history of non-MI CVD^a^	**Yes**	62	20	231	35	38	13	0.77	3.38	4.08
	**No**	26	123						2.10–5.45	2.47–6.76
Any parent or sibling with CVD before	**Yes**	126	25	243	62	56	47	0.77	1.84	1.44
age 65 ^b^	**No**	11	81						1.27–2.66	1.01–2.07
Prolonged sitting time at work	**Yes**	37	22	197	30	28	31	0.78	0.98	0.87
	**No**	18	120						0.62–1.56	0.54–1.20
Ever smoking	**Yes**	172	8	235	77	76	72	0.91	1.58	1.33
	**No**	2	53						1.04–2.38	0.89–1.98
Smokeless tobacco use	**Yes**	24	3	239	11	12	11	0.97	1.00	1.04
	**No**	4	208						0.54–1.85	1.58–1.89
Overweight	**Yes**	128	16	223	65	61	48	0.84	1.92	1.62
(BMI >25 kg/m^2^)	**No**	7	72						1.31–2.82	1.12–2.35
Obesity	**Yes**	18	8	223	12	13	12	0.92	1.01	1.10
(BMI >30 kg/m^2^)	**No**	10	187						0.57–1.78	0.64–1.87

OR-B based on data from cases and controls; OR-A based on data from proxy informants and controls; CI, confidence interval.

^a^CVD, Cardiovascular disease, includes any of four specific CVD diagnoses: heart failure, stroke, angina and intermittent claudication;

^b^Don’t know” answers considered missing.

Table 2 and S1 Table also show case-proxy two-way conditional tables and number of case-proxy pairs who gave information about each specific exposure. The median response proportion among case-proxy pairs in the 82 variables under study was 94% (n = 229), IQR 88%-98%. Among traditional cardiovascular exposures, the median response proportion was 97% (n = 235), IQR 92%-98%. The variable with the lowest response proportion among case-proxy pairs was hyperlipidaemia with 59% (n = 144) (Table 2). From the telephone interviews, performed to obtain exposure information where respondents had left questions unanswered, it was clear that in general the reason for leaving a question unanswered was lack of information.

### Disagreement between the cases and their spouses

**Fig 1:**
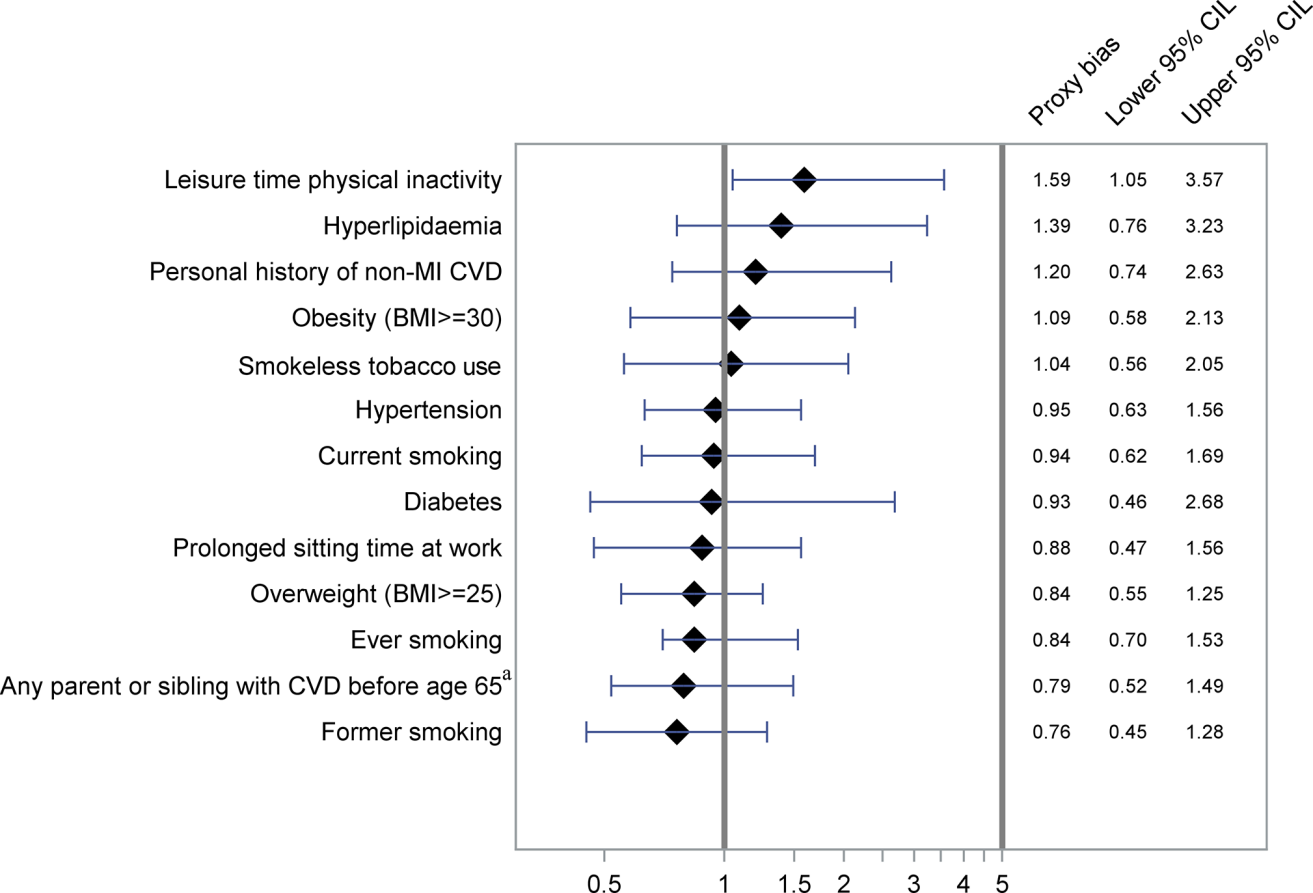
Proxy bias with 95% CI for traditional cardiovascular exposures. A proxy bias less than 1 indicates underestimation of the exposure by proxies compared to the cases. A proxy bias greater than 1 indicates overestimation of the exposure by proxies compared to the cases. CVD, Cardiovascular disease; CIL, Confidence Interval Limits. ^a^”Don’t know” answers considered unexposed.

**Fig 2:**
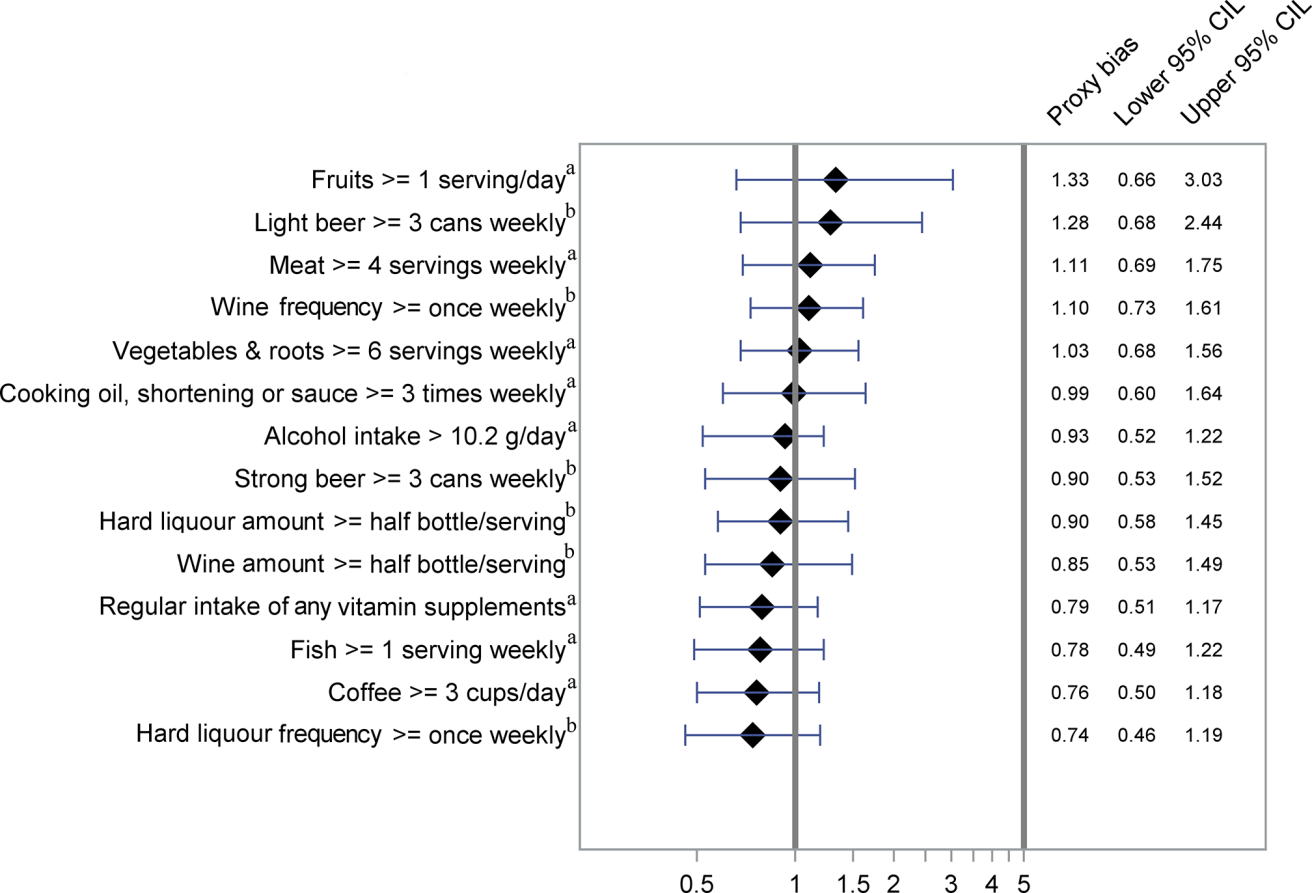
Proxy bias with 95% CI for dietary habits, intake of alcohol, coffee and vitamin supplements. A proxy bias less than 1 indicates underestimation of the exposure by proxies compared to the cases. A proxy bias greater than 1 indicates overestimation of the exposure by proxies compared to the cases CIL, Confidence Interval Limit. ^a^Reports pertaining to the one year-period preceding the myocardial infarction event; ^b^Reports pertaining to the 5–10 year-period preceding the myocardial infarction event.

**Fig 3:**
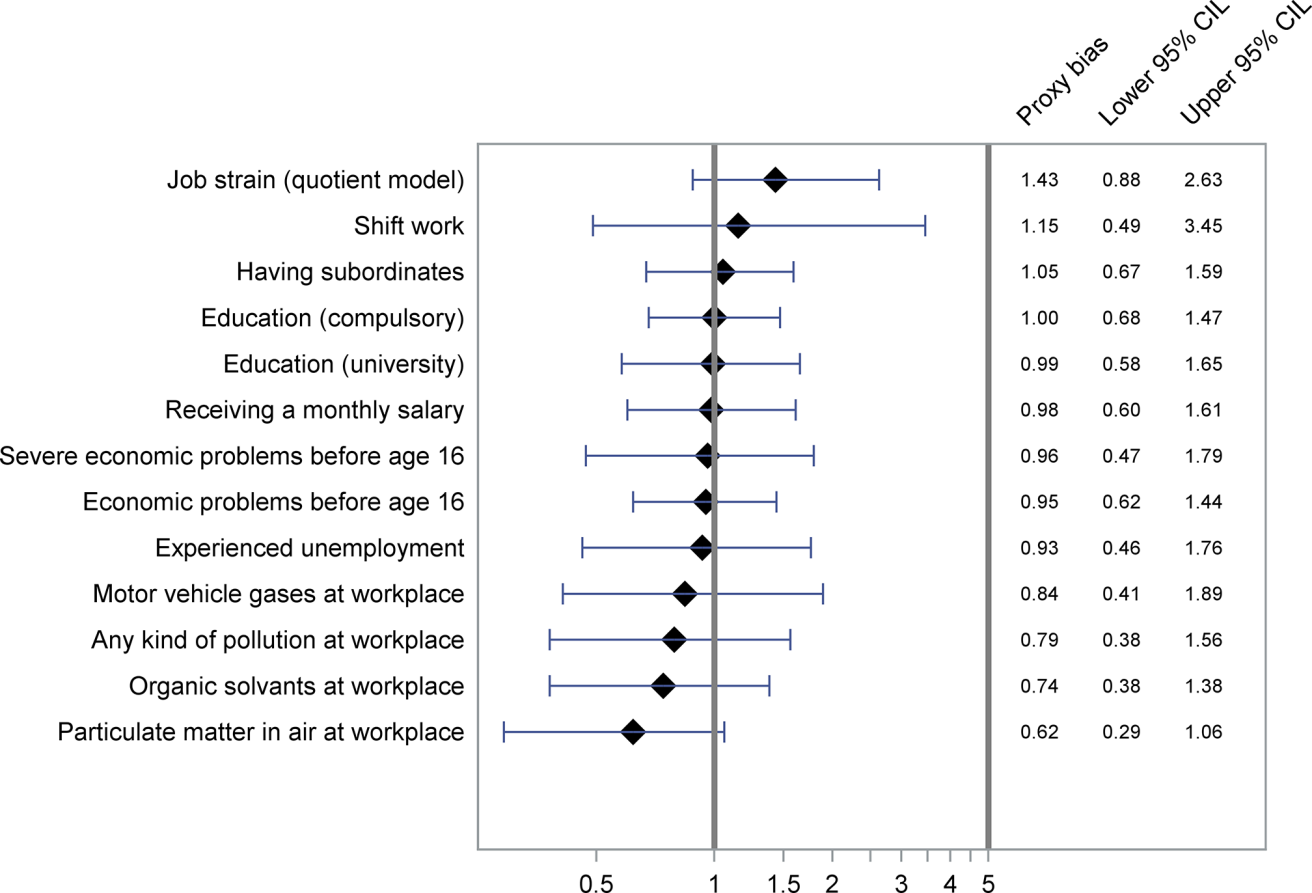
Proxy bias with 95% CI for work related factors. A proxy bias less than 1 indicates underestimation of the exposure by proxies compared to the cases. A proxy bias greater than 1 indicates overestimation of the exposure by proxies compared to the cases. CIL, Confidence Interval Limit. ^a^”Don’t know” answers considered unexposed.

**Fig 4:**
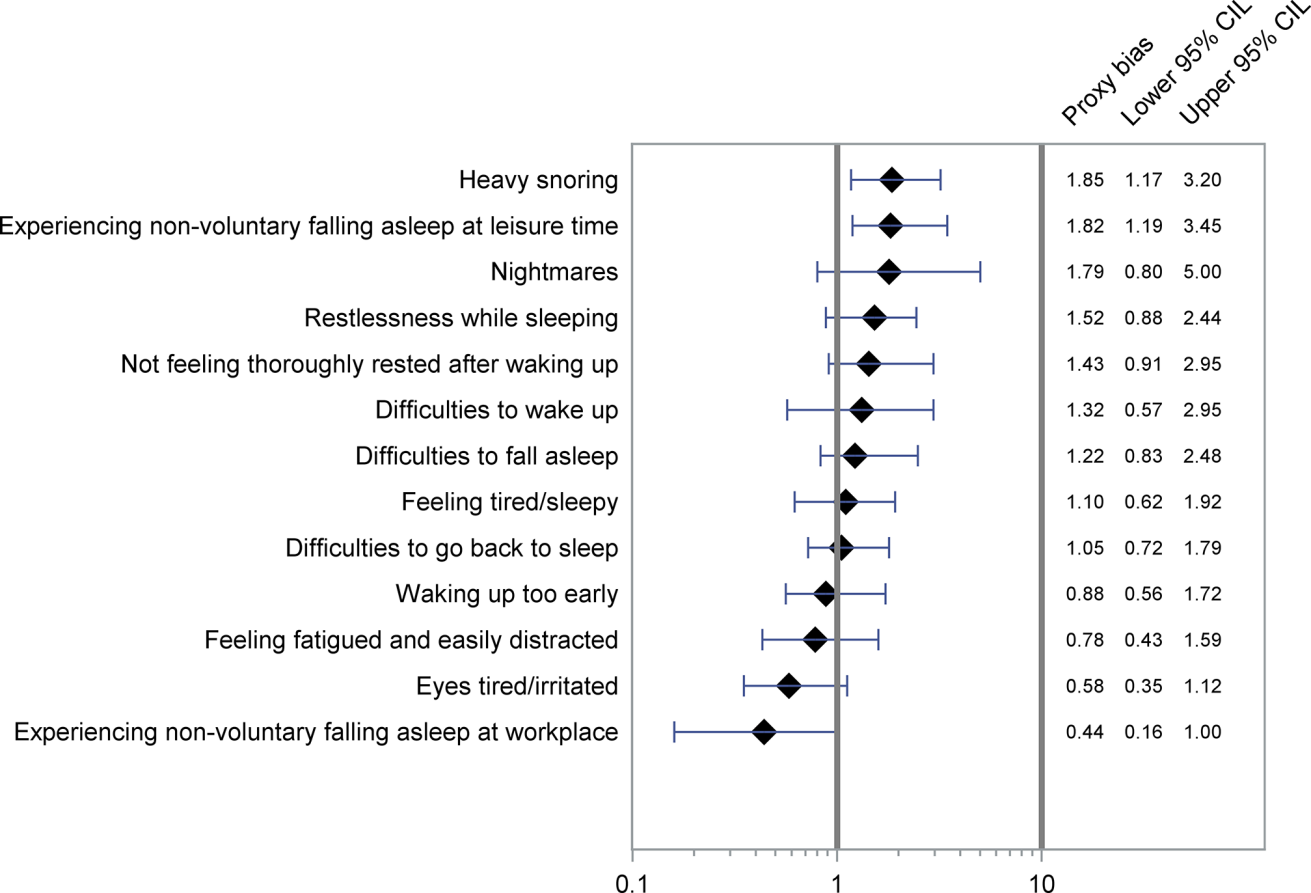
Proxy bias with 95% CI for sleep related problems. A proxy bias less than 1 indicates underestimation of the exposure by proxies compared to the cases. A proxy bias greater than 1 indicates overestimation of the exposure by proxies compared to the cases. CIL, Confidence Interval Limit.

The proxy biases along with 95% CI are shown in Figs 1–4 and S1 Fig and S2 Fig OR-A and OR-B with 95% CI forming basis for the calculations are shown in Table 2 and S1 Table, showing also kappa values for agreement between cases and proxies.

Traditional cardiovascular exposures. For a number of traditional cardiovascular risk factors, including hypertension, diabetes, hyperlipidaemia, ever smoking, obesity, and overweight, no clear discrepancies between data collected from cases and proxies were found (Fig 1). Similarly, for smokeless tobacco, we observed no significant discrepancy (Fig 1). Proxies, compared to cases, overestimated leisure time physical inactivity (proxy bias = 1.59, 95% CI 1.05–3.57), but proxies and cases reported similar estimates of prolonged sitting time at work (proxy bias = 0.88, 95% CI 0.47–1.56).

No clear discrepancy in the reporting was noted for a history of angina (S1 Fig). Reports of a history of other non-MI CVD were too few to produce any estimates. Pooled information from separate questions regarding history of heart failure, stroke, and intermittent claudication, termed personal history of non-MI CVD, showed no clear discrepancies (Fig 1).

No clear discrepancies between cases and proxies were found in the reporting of a history of CVD in the father or in the mother before the age of 65. These results hold irrespective of whether we excluded those who responded “don’t know” (S1 Fig) or whether we set them as unexposed. Using the latter approach, we found that the proxy bias for reporting CVD in the father was 0.92 (95% CI 0.74–1.79) and in the mother, 0.90 (95% CI 0.47–1.45). “Don’t know” answers regarding CVD in the father were reported by 15% of proxies and by 10% of cases. The respective figures for the mother and siblings were 18% and 10%, and 14% and 13%, respectively. Due to limited sample size, results reported in any sibling (S1 Fig), and in any close relative (Fig 1) are based on analyses where “don’t know” answers were reclassified as unexposed. For these variables, no clear discrepancies between reports from proxies and cases were observed.

Dietary habits, intake of alcohol, coffee and vitamin supplements. There were no major discrepancies between cases and proxies in their reporting of dietary habits (Fig 2). The frequency of coffee intake and the dietary supplement intake, however, tended to be underestimated by proxies proxy bias 0.76 (95% CI 0.50–1.18) and 0.79 (95% CI 0.51–1.17), respectively.

As shown in Fig 2, the use of proxy data did not introduce large discrepancies in the results obtained regarding frequent consumption of light beer, strong beer, and wine in the five- to ten-year period preceding the MI event. However, frequent consumption of hard liquor tended to be underestimated by the proxies (proxy bias = 0.74, 95% CI 0.46–1.19). There was no clear discrepancy between cases and their proxies in the reporting of large serving size of wine and hard liquor. A high average daily consumption of alcohol (>10.2 grams) in the preceding year was similarly established from reports of frequency and amount of alcohol intake by cases and proxies (proxy bias 0.93, 95% CI 0.52–1.22).

#### Work-related factors

We observed no large discrepancies in the reporting of indicators of socio-economic position (Fig 3). With regard to work-related exposures, we observed no large discrepancies in the reporting of shift work, having subordinates, and having a monthly salary. However, regarding reports of pollution at the workplace, the results indicate underestimation by proxies (proxy bias = 0.79, 95% CI 0.38–1.56). Because there were many “don’t know” answers (10% in cases and 29% in proxies) to the questions about pollution at workplace, we performed the analysis reclassifying “don’t know” answers as unexposed (proxy bias = 0.57, 95% CI 0.34–0.91). Job strain tended to be overestimated by proxies (proxy bias = 1.43, 95% CI 0.88–2.63). Results on non-voluntary falling asleep at workplace are reported below.

#### Psychosocial factors

The analyses of reports about important life events and coping strategies reveal no major discrepancies between the reports from proxies and cases (S2 Fig). However, the proxies tended to overestimate the frequency that cases felt things turned out the way he/she wanted (proxy bias = 1.59, 95% CI 0.95–2.78).

#### Sleep-related problems

In general, among the questions related to sleep, we observed no major discrepancies between reports from proxies and cases (Fig 4). However, for heavy snoring (proxy bias = 1.85, 95% CI 1.17–3.20), non-voluntary falling asleep during leisure time (proxy bias = 1.82, 95% CI 1.19–3.45), and non-voluntary falling asleep at workplace (proxy bias = 0.44, 95% CI 0.16–1.00), we observed gross discrepancies. Compared to the cases, proxies overestimated the frequency of the first two and underestimated the frequency of the latter (Fig 4).

## Discussion

The main finding of this study is that reports from cases and their spouses or common-law spouses on 82 of cardiovascular-related exposures in general do not show disagreement. However, as compared to index cases, proxies were observed to overestimate exposure to leisure time physical inactivity, non-voluntary falling asleep during leisure time and heavy snoring. As compared to cases, they also tended to overestimate the level of job strain and the frequency the index case felt things turned out the way he/she wanted. Compared to cases, proxies tended to underestimate the frequency of non-voluntary falling asleep at work, pollution at workplace, coffee consumption, and the intake of vitamin supplements.

The general absence of significant disagreement between cases and their spouses on the reporting of data on different exposures and characteristics observed in our study is also reported in earlier studies that evaluate the concordance between healthy index persons [4,5,13,14] and between non-MI CVD cases and their respective spouses [3,4]. The variables studied include body habitus [4], family [13] and personal history of CVD [3,6], smoking [3,4], highest education achievement [4,13], and alcohol consumption [3–5]. However, Capelle et al., studying index cases with neurological CVD (n = 30), report only moderate level of agreement between index cases and spouses for family history of CVD [3].

Among earlier studies investigating the validity of information collected from spouses of index cases, only Graham et al. included MI patients [7]. Graham et al. found fairly good agreement between MI cases and their spouses in the reporting of alcohol intake in the three months preceding the MI event. The daily amount of alcohol consumption was calculated in grams and analysed as a continuous variable [7]. Our findings, based on daily grams of intake, also show agreement between proxies and cases irrespective of whether we used a cut-off (Fig 3) or we used grams as a continuous variable (proxy bias 1.00, 95% CI 0.99–1.02). Our data, however, consider alcohol intake in the 12 months preceding the MI event. In regard to specific alcoholic beverage consumption in the five to ten years preceding the MI event, we observed that high hard liquor consumption tended to be underestimated by the proxies, whereas other types of alcohol beverages were similarly reported. Graham et al. reported no results on specific alcohol beverages.

It seems reasonable that some of the sleep-related exposures, in particular snoring, difficulty waking up, and falling asleep during leisure time, may be more accurately reported by proxies than by cases. In agreement with our findings of the discrepancy between cases and proxies in the reporting of some sleep related factors, Wiggins et al., comparing agreement between spouses (both of them healthy) living in New Mexico, report similar findings for snoring: the frequency of snoring reported by index men was lower than the frequency reported by their wives [15]. A similar trend was not observed in index women. Furthermore, Wiggins et al. report that women underestimate their husbands’ frequency of falling asleep at the workplace, but men do not make the same underestimation regarding their wives [15].

Surprisingly, the validity of proxy respondent reports of level of physical activity has rarely been studied. Nelson et al. report moderate level of discrepancies between index cases with subarachnoid haemorrhage and index controls and their respective spouses in the reporting of leisure time physical inactivity [4]. In our study, however, proxies grossly overestimated leisure time physical inactivity compared to index cases. In our study, the cases and proxies reported similar prolonged sitting time at work, an exposure not evaluated in earlier studies.

In our study, proxies compared to cases, tended to underestimate pollution at workplace. This result in combination with a considerably higher proportion of “don’t know” answers in proxies as compared to cases concerning these questions raises an issue around the usefulness of these data. A main strength of this study is the large number of cardiovascular risk exposures considered. Moreover, some of these exposures have not been considered in earlier studies that addressed level of agreement between different rates (patient-proxy pairs or pairs of healthy individuals): early familial economic problems, unemployment, working shifts, having subordinates, receiving a monthly salary, job strain, prolonged sitting time at work, use of smokeless tobacco and coping strategies.

In addition to contributing to knowledge about the possibilities of proxy data validity with respect to fatal cases in case-control studies of MI, our results might also contribute to increased knowledge in the clinical setting about the possibilities of receiving valid information from a spouse when the MI index case is unable to respond to questions about exposures and other characteristics of relevance for the diagnosis and subsequent treatment.

### Study limitations

A limitation of the present study is that it is restricted to spouses; other kinships may have different behaviours that prevent generalization of results to kinships other than spouses. Another limitation affecting the generalizability of results is that the education level of the spouse was not collected.

An important consideration when interpreting results is also the time that elapsed after cases filled in the questionnaire to the proxies had the chance to do so. The behaviours of some of the cases may have changed during this period.

Aiming to isolate the proxy biases, we must acknowledge that there is probably some level of misclassification of the different exposures in cases as well as in controls. Of note, the approach used does not allow us to elucidate the occurrence of such misclassifications. Thus, it is not clear as to what level our results could be generalized to other case-control studies where the misclassification of the exposures under study may not be the same as in our study. Although using exposure data reported by index cases is the gold standard for studies such as ours, it is relevant to speculate about whether the cases or the proxies reported the true level of exposure. In a fictive scenario where cases, proxies and controls do similar non-systematic misclassification, the proxy bias will deviate towards 1.0, thus indicating no disagreement between cases and proxies.

For a few variables among those we studied, our findings are inconclusive due to low prevalence.

## Conclusions

In general, our results suggest that spouses or common-law spouses and their respective index cases similarly report a majority of the cardiovascular traditional risk factors. However, compared to their index cases, proxies may differently report leisure time physical inactivity. Our study provides new insight regarding the usefulness of proxy data regarding a large number of exposures. Confirmatory studies are warranted.

## Supporting Information

S1 Fig Proxy bias with 95% CI for personal and family history of disease. A proxy bias less than 1 indicates underestimation of the exposure by proxies compared to the cases. A proxy bias greater than 1 indicates overestimation of the exposure by proxies compared to the cases. CVD, Cardiovascular disease; CHD, Coronary heart disease; CIL, Confidence Interval Limit; ^a^”Don’t know” answers considered unexposed; ^b^”Don’t know” answers considered missing.
(TIFF)

S2 Fig Proxy bias with 95% CI for psychosocial factors. A proxy bias less than 1 indicates underestimation of the exposure by proxies compared to the cases. A proxy bias greater than 1 indicates overestimation of the exposure by proxies compared to the cases. CIL, Confidence Interval Limit.
(TIFF)

S1 Dataset Raw collapsed data forming basis for optional calculations of additional agreement estimates. (XLSX)

S1 Tables Information forming basis for assessment of disagreement between cases’ and proxies’ reports. Table A, Information forming basis for assessment of disagreement between cases and proxies on the reporting of smoking habits. Table B, Information forming basis for assessment of disagreement between cases and proxies in the reporting of family history of cardiovascular disease. Table C, Information forming basis for assessment of disagreement between cases and proxies on the reporting of personal history of non-MI cardiovascular diseases (CVD). Table D, Information forming basis for assessment of disagreement between cases and proxies on reporting of dietary habits including coffee and vitamin supplement intake during the preceding year. Table E, Information forming basis for assessment of disagreement between cases and their proxies on the reporting of alcohol drinking habits. Table F, Information forming basis for assessment of disagreement between cases and proxies on the reporting of socioeconomic and work related factors. Table G, Information forming basis for assessment of disagreement between cases and their proxies on the reporting of important life events. Table H, Information forming basis for assessment of disagreement between cases and proxies on reporting of exposures related to coping strategies. Table I, Information forming basis for assessment of disagreement between cases and proxies on reporting of sleep-related problems.
(DOCX)
