# Supplementary figures and images for: Agreement between Myocardial Infarction Patients and Their Spouses on Reporting of Data on 82 Cardiovascular Risk Exposures
Source: PLoS One. 2015 Jul 10;10(7):e0132601. doi: 10.1371/journal.pone.0132601 (PMC4498787; doi:10.1371/journal.pone.0132601)

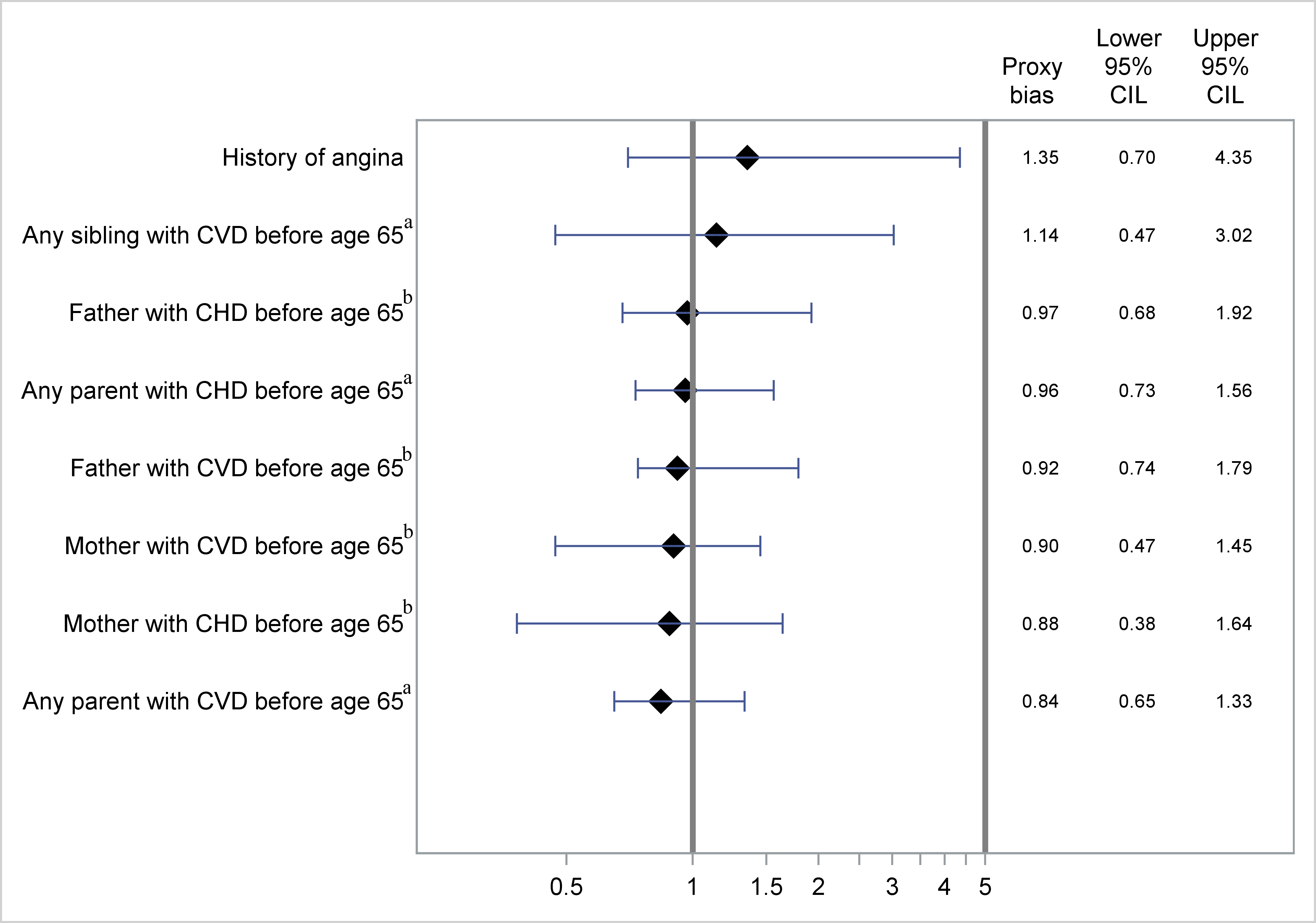

Supplement: S1 Fig — A proxy bias less than 1 indicates underestimation of the exposure by proxies compared to the cases. A proxy bias greater than 1 indicates overestimation of the exposure by proxies compared to the cases. CVD, Cardiovascular disease; CHD, Coronary heart disease; CIL, Confidence Interval Limit; a”Don’t know” answers considered unexposed; b”Don’t know” answers considered missing. (TIFF) [file pone.0132601.s001.tiff]

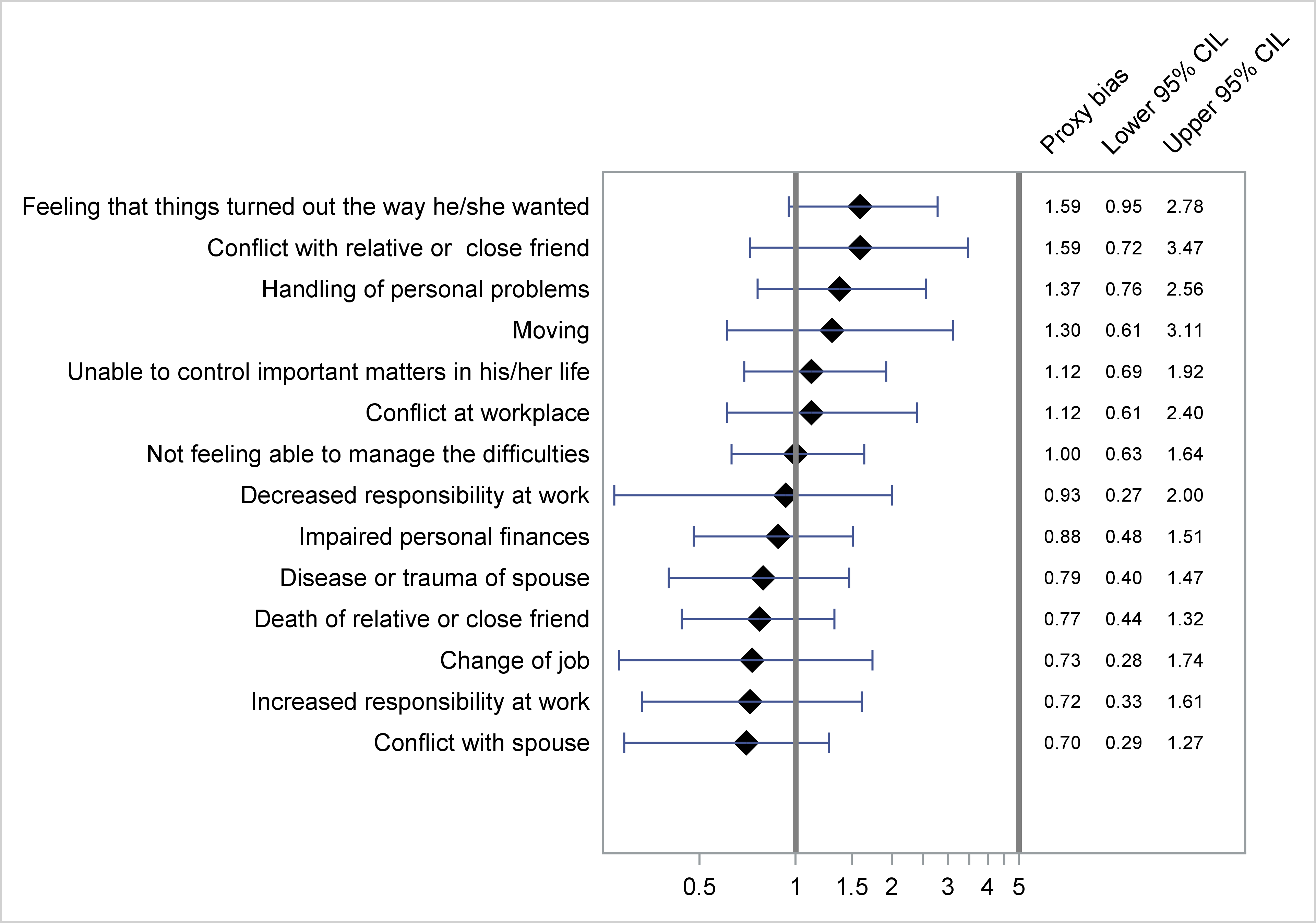

Supplement: S2 Fig — A proxy bias less than 1 indicates underestimation of the exposure by proxies compared to the cases. A proxy bias greater than 1 indicates overestimation of the exposure by proxies compared to the cases. CIL, Confidence Interval Limit. (TIFF) [file pone.0132601.s002.tiff]
