# Supplementary material for: Agreement between Myocardial Infarction Patients and Their Spouses on Reporting of Data on 82 Cardiovascular Risk Exposures
Source: PLoS One. 2015 Jul 10;10(7):e0132601. doi: 10.1371/journal.pone.0132601 (PMC4498787; doi:10.1371/journal.pone.0132601)
Supplement: S1 Tables — Table A, Information forming basis for assessment of disagreement between cases and proxies on the reporting of smoking habits. Table B, Information forming basis for assessment of disagreement between cases and proxies in the reporting of family history of cardiovascular disease. Table C, Information forming basis for assessment of disagreement between cases and proxies on the reporting of personal history of non-MI cardiovascular diseases (CVD). Table D, Information forming basis for assessment of disagreement between cases and proxies on reporting of dietary habits including coffee and vitamin supplement intake during the preceding year. Table E, Information forming basis for assessment of disagreement between cases and their proxies on the reporting of alcohol drinking habits. Table F, Information forming basis for assessment of disagreement between cases and proxies on the reporting of socioeconomic and work related factors. Table G, Information forming basis for assessment of disagreement between cases and their proxies on the reporting of important life events. Table H, Information forming basis for assessment of disagreement between cases and proxies on reporting of exposures related to coping strategies. Table I, Information forming basis for assessment of disagreement between cases and proxies on reporting of sleep-related problems. (DOCX) [file pone.0132601.s004.docx]

**S1 Tables**

**Table A. Information forming basis for assessment of disagreement between cases and proxies on the reporting of smoking habits.**

| **Variable** | **Case Response** | **Proxy Response** | | | **Number of pairs** | **Prevalence (%), case data** | **Prevalence (%), proxy data** | **Prevalence (%), control data** | **Kappa** | **OR-B** | **OR-A** |
| --- | --- | --- | --- | --- | --- | --- | --- | --- | --- | --- | --- |
|  |  | **Never** | **Current** | **Former** |  |  |  |  |  | **95% CI** | **95% CI** |
| Smoking | **Never** | 53 | 1 | 1 | 235 | 23 | 26 | 40 |  | 1 | 1 |
|  |  |  |  |  |  |  |  |  |  | Reference category | Reference category |
|  | **Current** | 8 | 56 | 8 |  | 46 | 48 | 30 |  | 2.48 | 2.35 |
|  |  |  |  |  |  |  |  |  |  | 2.11-3.97 | 1.46-3.73 |
|  | **Former** | 0 | 4 | 104 |  | 31 | 28 | 30 | 0.80 | 1.94 | 1.47 |
|  |  |  |  |  |  |  |  |  |  | 1.18-3.18 | 0.90-2.42 |

OR-B based on data from cases and controls; OR-A based on data from proxy informants and controls; CI, confidence interval.

**Table B. Information forming basis for assessment of disagreement between cases and proxies in the reporting of family history of cardiovascular disease.**

| **Variable** | **Case Response** | **Proxy Response** | | **Number of Pairs** | **Prevalence (%), case data** | **Prevalence (%), proxy data** | **Prevalence (%), control data** | **Kappa** | **OR-B** | **OR-A** |
| --- | --- | --- | --- | --- | --- | --- | --- | --- | --- | --- |
|  |  | **Yes** | **No** |  |  |  |  |  | **95% CI** | **95% CI** |
| Father with CHD before age 65^a^ | **Yes** | 51 | 12 | 190 | 33 | 32 | 28 | 0.87 | 1.22 | 1.18 |
|  | **No** | 10 | 117 |  |  |  |  |  | 0.80-1.85 | 0.76-1.83 |
| Father with CVD before age 65^a^ | **Yes** | 82 | 11 | 190 | 49 | 47 | 39 | 0.88 | 1.42 | 1.30 |
|  | **No** | 7 | 90 |  |  |  |  |  | 0.94-2.12 | 0.87-1.94 |
| Mother with CHD before age 65^a^ | **Yes** | 41 | 11 | 173 | 31 | 29 | 21 | 0.86 | 1.44 | 1.27 |
|  | **No** | 9 | 110 |  |  |  |  |  | 0.89-2.34 | 0.79-2.04 |
| Mother with CVD before age 65^a^ | **Yes** | 59 | 17 | 174 | 44 | 41 | 37 | 0.79 | 1.32 | 1.19 |
|  | **No** | 13 | 85 |  |  |  |  |  | 0.85-2.04 | 0.78-1.81 |
| Any parent with CHD before 65 years old^b^ | **Yes** | 101 | 19 | 243 | 49 | 49 | 37 | 0.80 | 1.61 | 1.55 |
|  | **No** | 17 | 106 |  |  |  |  |  | 1.13-2.25 | 1.09-2.22 |
| Any parent with CVD before 65 years old^b^ | **Yes** | 116 | 20 | 243 | 56 | 51 | 43 | 0.83 | 1.59 | 1.33 |
|  | **No** | 9 | 98 |  |  |  |  |  | 1.12-2.25 | 0.94-1.89 |
| Any sibling with CVD before 65 years old^b^ | **Yes** | 28 | 8 | 243 | 16 | 16 | 11 | 0.93 | 1.47 | 1.58 |
|  | **No** | 10 | 197 |  |  |  |  |  | 0.82-2.64 | 0.89-2.80 |

CHD, Coronary heart disease; CVD, Cardiovascular disease; OR-B based on data from cases and controls; OR-A based on data from proxy informants and controls; CI, confidence interval.

^a^ “Don’t know” answers considered missing.

^b^ “Don’t know” answers considered unexposed.

**Table C. Information forming basis for assessment of disagreement between cases and proxies on the reporting of personal history of non-MI cardiovascular diseases (CVD).**

| **Variable** | **Case Response** | **Proxy Response** | | **Number of pairs** | **Prevalence (%), case data** | **Prevalence (%), proxy data** | **Prevalence (%), control data** | **Kappa** | **OR-B** | **OR-A** |
| --- | --- | --- | --- | --- | --- | --- | --- | --- | --- | --- |
|  |  | **Yes** | **No** |  |  |  |  |  | **95% CI** | **95% CI** |
| Angina | **Yes** | 46 | 29 | 229 | 28 | 33 | 8 | 0.77 | 5.00 | 6.75 |
|  | **No** | 18 | 136 |  |  |  |  |  | 2.68-9.31 | 3.48-13.12 |
| Stroke^a^ | **Yes** | 3 | 0 | 241 | 2 | 1 | 2 | 0.75 | 0.83 | 0.50 |
|  | **No** | 2 | 236 |  |  |  |  |  | 0.25-2.73 | 0.12-2.00 |
| Intermittent claudication^a^ | **Yes** | 12 | 12 | 236 | 8 | 10 | 5 | 0.92 | 1.52 | 2.14 |
|  | **No** | 6 | 206 |  |  |  |  |  | 0.72-3.21 | 1.03-4.43 |
| Heart failure^a^ | **Yes** | 4 | 13 | 235 | 4 | 7 | 2 | 0.95 | 2.39 | 5.57 |
|  | **No** | 5 | 213 |  |  |  |  |  | 0.72-7.79 | 1.14-19.20 |

OR-B based on data from cases and controls; OR-A based on data from proxy informants and controls; CI, confidence interval.

^a^The bootstrap analysis was inefficient and no stable results could be obtained.

**Table D. Information forming basis for assessment of disagreement between cases and proxies on reporting of dietary habits including coffee and vitamin supplement intake during the preceding year.**

| **Variable** | **Case Response** | **Proxy Response** | | **Number of pairs** | **Prevalence (%), case data** | **Prevalence (%), proxy data** | **Prevalence (%), control data** | **Kappa** | **OR-B** | **OR-A** |
| --- | --- | --- | --- | --- | --- | --- | --- | --- | --- | --- |
|  |  | **Yes** | **No** |  |  |  |  |  | **95% CI** | **95% CI** |
| Fruit (the median or above:≥ 1 serving daily) | **Yes** | 9 | 17 | 238 | 11 | 14 | 16 | 0.82 | 0.64 | 0.86 |
|  | **No** | 25 | 187 |  |  |  |  |  | 0.38-1.10 | 0.51-1.43 |
| Vegetables and roots (the median or above:≥ 6 weekly servings) | **Yes** | 70 | 38 | 237 | 46 | 47 | 51 | 0.56 | 0.82 | 0.84 |
|  | **No** | 41 | 88 |  |  |  |  |  | 0.58-1.15 | 0.59-1.20 |
| Meat (the median or above: ≥ 4 weekly servings) | **Yes** | 69 | 43 | 239 | 47 | 50 | 43 | 0.49 | 1.21 | 1.34 |
|  | **No** | 51 | 76 |  |  |  |  |  | 0.84-1.74 | 0.94-1.92 |
| Fish (the median or above: ≥ 1 weekly servings) | **Yes** | 70 | 47 | 238 | 49 | 43 | 43 | 0.58 | 1.25 | 0.98 |
|  | **No** | 32 | 89 |  |  |  |  |  | 0.87-1.79 | 0.68-1.40 |
| Fat, shortening and sauce (the median or above: ≥ 3 weekly servings) | **Yes** | 72 | 49 | 237 | 51 | 50 | 42 | 0.45 | 1.56 | 1.54 |
|  | **No** | 47 | 69 |  |  |  |  |  | 1.07-2.27 | 1.05-2.27 |
| Coffee intake (the median or above: ≥ 3 daily cups) | **Yes** | 112 | 30 | 237 | 60 | 53 | 45 | 0.72 | 1.86 | 1.41 |
|  | **No** | 14 | 81 |  |  |  |  |  | 1.27-2.70 | 0.97-2.04 |
| Regular intake of any vitamin supplements | **Yes** | 56 | 25 | 240 | 34 | 28 | 37 | 0.83 | 0.90 | 0.71 |
|  | **No** | 11 | 148 |  |  |  |  |  | 0.63-1.27 | 0.49-1.01 |

OR-B based on data from cases and controls; OR-A based on data from proxy informants and controls; CI, confidence interval.

**Table E. Information forming basis for assessment of disagreement between cases and their proxies on the reporting of alcohol drinking habits.**

| **Variable** | **Case Response** | **Proxy Response** | | **Number of Pairs** | **Prevalence (%), case data** | **Prevalence (%), proxy data** | **Prevalence (%), control data** | **Kappa** | **OR-B** | **OR-A** |
| --- | --- | --- | --- | --- | --- | --- | --- | --- | --- | --- |
|  |  | **Yes** | **No** |  |  |  |  |  | **95% CI** | **95% CI** |
| Light beer ( the median or above: ≥ 3 cans or bottles/week)^a^ | **Yes** | 28 | 14 | 203 | 21 | 24 | 18 | 0.82 | 1.17 | 1.50 |
|  | **No** | 21 | 140 |  |  |  |  |  | 0.70-1.96 | 0.90-2.53 |
| Strong beer (the median or above: ≥ 3 cans or bottles/week)^a^ | **Yes** | 41 | 24 | 207 | 31 | 29 | 36 | 0.77 | 0.81 | 0.73 |
|  | **No** | 20 | 122 |  |  |  |  |  | 0.53-1.25 | 0.48-1.12 |
| Wine frequency (the median or above: ≥ once weekly)^a^ | **Yes** | 92 | 12 | 217 | 48 | 50 | 48 | 0.83 | 1.03 | 1.40 |
|  | **No** | 17 | 96 |  |  |  |  |  | 0.72-1.48 | 0.79-1.64 |
| Wine amount (the median or above: ≥ half a bottle)^a^ | **Yes** | 42 | 27 | 214 | 32 | 29 | 37 | 0.76 | 0.80 | 0.69 |
|  | **No** | 20 | 125 |  |  |  |  |  | 0.53-1.22 | 0.45-1.04 |
| Hard liquor frequency (the median or above: ≥ once weekly)^a^ | **Yes** | 53 | 30 | 216 | 38 | 31 | 40 | 0.77 | 0.93 | 0.69 |
|  | **No** | 14 | 119 |  |  |  |  |  | 0.62-1.38 | 0.46-1.02 |
| Hard liquor amount (the median or above: ≥ half a bottle)^a^ | **Yes** | 91 | 27 | 214 | 55 | 53 | 64 | 0.67 | 0.72 | 0.65 |
|  | **No** | 23 | 73 |  |  |  |  |  | 0.49-1.06 | 0.44-097 |
| High alcohol intake (above the median: >10.2 g/day)^b^ | **Yes** | 80 | 28 | 240 | 45 | 43 | 52 | 0.73 | 0.76 | 0.71 |
|  | **No** | 24 | 108 |  |  |  |  |  | 0.53-1.11 | 0.49-1.024 |

OR-B based on data from cases and controls; OR-A based on data from proxy informants and controls; CI, confidence interval.

^a^Based on reports during the 5-10 years preceding the myocardial infarction event.

^b^Based on reports during a year preceding the myocardial infarction event.

**Table F. Information forming basis for assessment of disagreement between cases and proxies on the reporting of socioeconomic and work related factors.**

| **Variable** | **Case Response** | **Proxy Response** | | **Number of pairs** | **Prevalence (%), case data** | **Prevalence (%), proxy data** | **Prevalence (%), control data** | **Kappa** | **OR-B** | **OR-A** |
| --- | --- | --- | --- | --- | --- | --- | --- | --- | --- | --- |
|  |  | **Yes** | **No** |  |  |  |  |  | **95% CI** | **95% CI** |
| Education (compulsory) | **Yes** | 108 | 16 | 238 | 52 | 52 | 47 | 0.82 | 1.19 | 1.19 |
|  | **No** | 16 | 98 |  |  |  |  |  | 0.84-1.70 | 0.84-1.70 |
| Education (university) | **Yes** | 33 | 5 | 238 | 16 | 16 | 20 | 0.96 | 0.78 | 0.78 |
|  | **No** | 5 | 195 |  |  |  |  |  | 0.49-1.62 | 0.48-1.62 |
| Economic problems before age 16 | **Yes** | 74 | 29 | 223 | 46 | 45 | 52 | 0.68 | 0.82 | 0.78 |
|  | **No** | 26 | 94 |  |  |  |  |  | 0.57-1.17 | 0.54-1.10 |
| Severe economic problems before age 16 | **Yes** | 17 | 16 | 223 | 15 | 14 | 18 | 0.86 | 0.79 | 0.76 |
|  | **No** | 15 | 175 |  |  |  |  |  | 0.48-1.30 | 0.46-1.27 |
| Job strain (quadrant model)^a^ | **Yes** | 2 | 7 | 224 | 4 | 11 | 3 | 0.87 | 1.26 | 3.00 |
|  | **No** | 22 | 193 |  |  |  |  |  | 0.42-3.81 | 1.34-6.68 |
| Job Strain (quotient model) | **Yes** | 35 | 24 | 224 | 26 | 34 | 23 | 0.68 | 1.22 | 1.75 |
|  | **No** | 42 | 123 |  |  |  |  |  | 0.78-1.93 | 1.16-2.66 |
| Receiving a monthly salary | **Yes** | 173 | 14 | 237 | 79 | 78 | 77 | 0.71 | 1.09 | 1.07 |
|  | **No** | 13 | 37 |  |  |  |  |  | 0.70-1.70 | 0.70-1.65 |
| Shift work | **Yes** | 12 | 6 | 236 | 8 | 9 | 6 | 0.94 | 1.54 | 1.76 |
|  | **No** | 8 | 212 |  |  |  |  |  | 0.75-3.16 | 0.85-3.66 |
| Having subordinates | **Yes** | 79 | 18 | 238 | 41 | 42 | 44 | 0.81 | 0.85 | 0.90 |
|  | **No** | 20 | 121 |  |  |  |  |  | 0.58-1.25 | 0.62-1.30 |
| Experience unemployment | **Yes** | 15 | 11 | 234 | 11 | 10 | 15 | 0.91 | 0.79 | 0.74 |
|  | **No** | 9 | 199 |  |  |  |  |  | 0.46-1.36 | 0.43-1.26 |
| Motor vehicle gases at workplace | **Yes** | 22 | 13 | 197 | 18 | 15 | 13 | 0.89 | 1.52 | 1.28 |
|  | **No** | 8 | 154 |  |  |  |  |  | 0.86-2.69 | 0.69-2.38 |
| Particulate matter in air at workplace | **Yes** | 9 | 18 | 196 | 14 | 10 | 10 | 0.85 | 1.59 | 1.13 |
|  | **No** | 11 | 158 |  |  |  |  |  | 0.83-3.06 | 0.57-2.24 |
| Organic solvents at workplace | **Yes** | 25 | 15 | 194 | 21 | 16 | 18 | 0.89 | 1.22 | 0.90 |
|  | **No** | 7 | 147 |  |  |  |  |  | 0.71-2.11 | 0.53-1.52 |
| Lead pollution at workplace^a^ | **Yes** | 4 | 6 | 210 | 5 | 4 | 7 | 0.95 | 0.68 | 0.58 |
|  | **No** | 5 | 195 |  |  |  |  |  | 0.31-1.52 | 0.24-1.37 |
| Use of dynamite at workplace^a^ | **Yes** | 1 | 4 | 235 | 2 | 0 | 3 | 0.33 | 0.62 | 0.12 |
|  | **No** | 0 | 230 |  |  |  |  |  | 0.20-1.91 | 0.02-1.00 |
| Any kind of pollution at workplace^b^ | **Yes** | 55 | 35 | 151 | 60 | 56 | 37 | 0.35 | 0.65 | 0.52 |
|  | **No** | 30 | 31 |  |  |  |  |  | 0.41-1.03 | 0.31-0.86 |

OR-B based on data from cases and controls; OR-A based on data from proxy informants and controls; CI, confidence interval.

^a^The bootstrap analysis was inefficient and no stable results could be obtained.

^b^ “Don’t know” answers considered missing.

**Table G. Information forming basis for assessment of disagreement between cases and their proxies on the reporting of important life events.**

| **Variable** | **Case Response** | **Proxy Response** | | **Number of pairs** | **Prevalence (%), case data** | **Prevalence (%), proxy data** | **Prevalence (%), control data** | **Kappa** | **OR-B** | **OR-A** |
| --- | --- | --- | --- | --- | --- | --- | --- | --- | --- | --- |
|  |  | **Yes** | **No** |  |  |  |  |  | **95% CI** | **95% CI** |
| Conflict with spouse | **Yes** | 17 | 22 | 238 | 16 | 13 | 19 | 0.85 | 0.82 | 0.58 |
|  | **No** | 13 | 186 |  |  |  |  |  | 0.50-1.36 | 0.33-1.00 |
| Conflict with relative or close friend | **Yes** | 6 | 13 | 234 | 8 | 12 | 11 | 0.85 | 0.69 | 1.09 |
|  | **No** | 23 | 192 |  |  |  |  |  | 0.37-1.28 | 0.62-1.90 |
| Disease or trauma of spouse | **Yes** | 15 | 17 | 237 | 14 | 11 | 15 | 0.88 | 0.88 | 0.70 |
|  | **No** | 11 | 194 |  |  |  |  |  | 0.54-1.44 | 0.42-1.18 |
| Death of relative or close friend | **Yes** | 32 | 31 | 236 | 27 | 22 | 24 | 0.77 | 1.10 | 0.85 |
|  | **No** | 20 | 153 |  |  |  |  |  | 0.73-1.66 | 0.55-1.31 |
| Impaired personal finances | **Yes** | 27 | 17 | 201 | 19 | 16 | 19 | 0.88 | 1.00 | 0.87 |
|  | **No** | 12 | 181 |  |  |  |  |  | 0.63-1.57 | 0.54-1.39 |
| Conflict at workplace | **Yes** | 24 | 21 | 206 | 20 | 21 | 11 | 0.79 | 2.15 | 2.42 |
|  | **No** | 24 | 156 |  |  |  |  |  | 1.26-3.67 | 1.39-4.20 |
| Moving | **Yes** | 12 | 6 | 237 | 8 | 10 | 8 | 0.93 | 1.00 | 1.30 |
|  | **No** | 11 | 208 |  |  |  |  |  | 0.52-1.93 | 0.69-2.44 |
| Change of job | **Yes** | 10 | 13 | 228 | 10 | 8 | 10 | 0.91 | 0.99 | 0.73 |
|  | **No** | 8 | 197 |  |  |  |  |  | 0.55-1.78 | 0.37-1.41 |
| Decreased responsibility at work | **Yes** | 6 | 13 | 223 | 9 | 8 | 8 | 0.89 | 1.04 | 0.96 |
|  | **No** | 12 | 192 |  |  |  |  |  | 0.54-1.98 | 0.51-1.84 |
| Increased responsibility at work | **Yes** | 16 | 11 | 220 | 17 | 13 | 14 | 0.85 | 1.29 | 0.93 |
|  | **No** | 13 | 170 |  |  |  |  |  | 0.74-2.23 | 0.52-1.67 |

OR-B based on data from cases and controls; OR-A based on data from proxy informants and controls; CI, confidence interval.

**Table H. Information forming basis for assessment of disagreement between cases and proxies on reporting of exposures related to coping strategies.**

| **Variable** | **Case Response** | **Proxy Response** | | **Number of pairs** | **Prevalence (%), case data** | **Prevalence (%), proxy data** | **Prevalence (%), control data** | **Kappa** | **OR-B** | **OR-A** |
| --- | --- | --- | --- | --- | --- | --- | --- | --- | --- | --- |
|  |  | **Yes** | **No** |  |  |  |  |  | **95% CI** | **95% CI** |
| Unable to control important matters in his/her life | **Yes** | 90 | 46 | 223 | 61 | 64 | 57 | 0.28 | 1.14 | 1.28 |
|  | **No** | 52 | 35 |  |  |  |  |  | 0.77-1.66 | 0.88-1.86 |
| Handling of personal problems | **Yes** | 18 | 27 | 226 | 20 | 25 | 20 | 0.69 | 0.99 | 1.35 |
|  | **No** | 38 | 143 |  |  |  |  |  | 0.63-1.56 | 0.84-2.15 |
| Feeling that things turn out the way he/she wanted | **Yes** | 29 | 23 | 223 | 23 | 33 | 26 | 0.67 | 0.91 | 1.44 |
|  | **No** | 45 | 126 |  |  |  |  |  | 0.58-1.42 | 0.97-2.13 |
| Not feeling able to manage the difficulties | **Yes** | 101 | 38 | 229 | 61 | 61 | 67 | 0.48 | 0.78 | 0.77 |
|  | **No** | 38 | 52 |  |  |  |  |  | 0.53-1.12 | 0.53-1.12 |

OR-B based on data from cases and controls; OR-A based on data from proxy informants and controls; CI, confidence interval.

**Table I. Information forming basis for assessment of disagreement between cases and proxies on reporting of sleep-related problems.**

| **Variable** | **Case Response** | **Proxy Response** | | **Number of pairs** | **Prevalence (%), case data** | **Prevalence (%), proxy data** | **Prevalence (%), control data** | **Kappa** | **OR-B** | **OR-A** |
| --- | --- | --- | --- | --- | --- | --- | --- | --- | --- | --- |
|  |  | **Yes** | **No** |  |  |  |  |  | **95% CI** | **95% CI** |
| Difficulties to fall sleep | **Yes** | 64 | 24 | 235 | 37 | 42 | 32 | 0.70 | 1.33 | 1.63 |
|  | **No** | 34 | 113 |  |  |  |  |  | 0.90-1.96 | 1.10-2.42 |
| Difficulties to wake up | **Yes** | 18 | 20 | 234 | 16 | 19 | 14 | 0.79 | 1.39 | 1.83 |
|  | **No** | 27 | 169 |  |  |  |  |  | 0.78-2.45 | 1.04-3.24 |
| Difficulties to go back to sleep | **Yes** | 63 | 29 | 228 | 40 | 41 | 33 | 0.69 | 1.39 | 1.47 |
|  | **No** | 31 | 105 |  |  |  |  |  | 0.96-2.02 | 1.00-2.16 |
| Heavy snoring | **Yes** | 106 | 16 | 235 | 52 | 66 | 43 | 0.59 | 1.43 | 2.64 |
|  | **No** | 50 | 63 |  |  |  |  |  | 1.00-2.04 | 1.79-3.89 |
| Nightmares | **Yes** | 10 | 16 | 207 | 13 | 19 | 13 | 0.77 | 0.91 | 1.62 |
|  | **No** | 30 | 151 |  |  |  |  |  | 0.51-1.62 | 0.91-2.88 |
| Not feeling thoroughly rested after waking up | **Yes** | 71 | 32 | 228 | 45 | 53 | 50 | 0.52 | 0.83 | 1.18 |
|  | **No** | 49 | 76 |  |  |  |  |  | 0.56-1.22 | 0.80-1.73 |
| Waking up too early | **Yes** | 44 | 47 | 231 | 39 | 36 | 39 | 0.56 | 1.03 | 0.91 |
|  | **No** | 40 | 100 |  |  |  |  |  | 0.71-1.50 | 0.63-1.32 |
| Restlessness while sleeping | **Yes** | 49 | 23 | 230 | 31 | 41 | 36 | 0.66 | 0.86 | 1.32 |
|  | **No** | 46 | 112 |  |  |  |  |  | 0.59-1.26 | 0.91-1.90 |
| Feeling tired/sleepy | **Yes** | 83 | 38 | 229 | 53 | 54 | 41 | 0.52 | 1.69 | 1.86 |
|  | **No** | 41 | 67 |  |  |  |  |  | 1.15-2.48 | 1.25-2.77 |
| Eyes tired/irritated | **Yes** | 38 | 54 | 214 | 43 | 30 | 32 | 0.58 | 1.60 | 0.93 |
|  | **No** | 26 | 96 |  |  |  |  |  | 1.08-2.36 | 0.60-1.42 |
| Experiencing non-voluntary periods of sleep at work | **Yes** | 9 | 35 | 195 | 23 | 12 | 22 | 0.74 | 1.13 | 0.50 |
|  | **No** | 14 | 137 |  |  |  |  |  | 0.69-1.87 | 0.28-0.88 |
| Experiencing non-voluntary falling asleep during leisure time | **Yes** | 58 | 25 | 235 | 35 | 48 | 32 | 0.59 | 1.20 | 2.18 |
|  | **No** | 54 | 98 |  |  |  |  |  | 0.82-1.74 | 1.45-3.27 |
| Feeling fatigued and easily distracted | **Yes** | 40 | 43 | 210 | 40 | 35 | 33 | 0.57 | 1.44 | 1.12 |
|  | **No** | 34 | 93 |  |  |  |  |  | 0.93-2.21 | 0.75-1.65 |

OR-B based on data from cases and controls; OR-A based on data from proxy informants and controls; CI, confidence interval.
